# Genetic Variability in Mediterranean Coastal Ecosystems: Insights into *Ostrea* spp. (Bivalvia: Ostreidae)

**DOI:** 10.3390/biology13090702

**Published:** 2024-09-07

**Authors:** Giuseppe Esposito, Simone Peletto, Ximing Guo, Paolo Pastorino, Alessandra Arillo, Isabella Martini, Pier Luigi Acutis, Davide Mugetti, Domenico Meloni, Marino Prearo, Paola Modesto

**Affiliations:** 1 Istituto Zooprofilattico Sperimentale del Piemonte, Liguria e Valle d’Aosta, 10154 Turin, Italy; simone.peletto@izsto.it (S.P.); isabella.martini@izsto.it (I.M.); pierluigi.acutis@izsto.it (P.L.A.); marino.prearo@izsto.it (M.P.);; 2 Haskin Shellfish Research Laboratory, Department of Marine and Coastal Sciences, Rutgers University, Port Norris, NJ 08349, USA; xguo@hsrl.rutgers.edu; 3 Department of Veterinary Medicine, 07100 Sassari, Italy; dmeloni@uniss.it

**Keywords:** bivalve mollusc, coastal lagoon, biodiversity, 16S rRNA, fisheries and aquaculture

## Abstract

**Simple Summary:**

Oysters are sessile, filter-feeding bivalves that are vital to fisheries and ecology but are poorly understood in terms of genetic diversity and distribution. This study examines small flat oysters in the Mediterranean regions of Liguria and Sardinia (Italy) using 16S rRNA sequencing to identify species. The findings reveal new insights into the *Ostrea stentina* complex and introduce a new species, *O. neostentina*, improving knowledge of *Ostrea* species diversity and distribution.

**Abstract:**

Oysters are sessile, filter-feeding bivalve molluscs widely distributed in estuarine and coastal habitats worldwide. They constitute a valuable resource for fisheries and extensive aquaculture and provide essential ecological services. Yet, their genetic diversity and distribution remain understudied. The variability in shell morphology complicates species classification, which is influenced by environmental and genetic factors. Although molecular phylogenetics research has refined oyster taxonomy and identified approximately 100 extant species, numerous taxonomic uncertainties persist. In the present study, we aimed to document the occurrence of small flat oysters of the genus *Ostrea* along the Mediterranean coastal areas of Liguria and Sardinia (Italy). Specifically, 16S rRNA sequence data were used to identify *Ostrea* species. Our findings offer novel insights into the *O. stentina* species complex and *O. neostentina*, a new species in the Mediterranean coastal areas of Italy. The study data further our understanding of *Ostrea* species diversity, distribution, and evolutionary patterns.

## 1. Introduction

Oysters are sessile, filter-feeding bivalve molluscs widely distributed in estuarine and coastal habitats worldwide. They provide resources for fisheries and extensive aquaculture, as well as important ecological services [1,2]. Oyster farming plays a crucial role in aquaculture [3,4] as a popular food source [5,6,7] in many countries [8].

Most marine bivalve production (90.7% or 16,551,342 tons live weight) comes from aquaculture, with only 9.3% (1,533,597 tons) from fisheries. In 2021, the Ostreidae family contributed 3.8% (6,802,582 tons) to global production, which totaled approximately 182 million tons from both capture and aquaculture [9]. Within global molluscan shellfish production (18,084,939 tons), oysters represented a significant portion at 38.6% [9].

A combination of overfishing, habitat depletion, water contamination, disease outbreaks, and lack of adequate management plans has led to serious population declines of the native European flat oyster, *Ostrea edulis* [1,2,10,11]. To offset the decline of *O. edulis* populations, the non-indigenous Pacific cupped oyster, *Crassostrea gigas* (also proposed as *Magallana gigas*; [12]), native to Japan and East Asia [9], was introduced throughout Europe and the Mediterranean [13,14].

Shellfish farming in the Mediterranean (over 640,000 hectares of nearly 400 wet environments) had a bivalve production of more than 800,000 tons in 2021 [9]. Oysters alone accounted for 55.8% (114,531 tons) of total European shellfish production, mainly attributable to *C. gigas* (98.5%; 112,850 tons) [9]. *C. gigas* is one of the most extensively cultivated oysters worldwide. Nearly ubiquitous [8,15], the species has gained hold of numerous wild habitats in regions where it was initially introduced, and wild populations are now established [8,16].

Several oyster species belonging to the genera *Ostrea* and *Crassostrea* have been identified along the Mediterranean coast [4]. Genetic analysis of *O. edulis* along the European Mediterranean coast has revealed significant regional differences and isolation by distance, indicating the presence of distinct local stocks rather than a single panmictic population [17]. This genetic differentiation highlights the importance of careful management in translocation and genetic enhancement programs to maintain wild genetic diversity [18,19]. 

Small flat oysters of the genus *Ostrea*, within the *O. stentina* species complex, are small, oval, or round, with height usually greater than length. The left valves have raised margins and shallow umbonal cavities, while the right valves are flatter with narrow commissural shelves. Both valves have fine crenelations along the right valve’s ligament, and their interiors range from dull gray to green, with a pointed, reniform adductor muscle scar surrounded by a greenish hue.

While hybridisation and introgression can pose risks, they can also benefit aquaculture by improving lipid nutritional quality, stress resistance, growth rates, and meat quality [20,21,22,23]. However, the genetic diversity and distribution of oysters remain underexplored.

The plasticity of shell morphology affects species classification, as shell differences result from environmental and genetic factors [24]. While population genetics studies have greatly improved oyster taxonomy, and roughly 100 living species are now recognized [25,26], numerous taxonomic uncertainties persist.

To address the controversies surrounding taxonomic and species diversity within the genus *Ostrea*, it is essential to provide context on the number of currently recognized species and the taxonomic history of the *O. stentina* species complex. Specifically, the *O. stentina* complex has undergone several revisions: initially classified under the genus *Ostreola*, it was later reverted to *Ostrea* due to a lack of distinctive morphological characteristics and genetic support [27,28,29,30,31,32]. Recent studies have suggested that several species, such as *O. equestris* and *O. aupouria*, may be synonyms of *O. stentina* or represent separate entities, highlighting the need for further taxonomic clarification [12,33,34].

Here, we report on small flat oysters of the genus *Ostrea* in two areas (Liguria and Sardinia) on the Italian Mediterranean coast. *Ostrea* species were identified through sequencing of the 16S rRNA gene. The present study provides a comprehensive update on the *O. stentina* species complex and on the novel *O. neostentina* in the Mediterranean coastal areas of Italy. Furthermore, our data help further our understanding of the genetic variability and distribution of *Ostrea* species.

## 2. Materials and Methods

### 2.1. Study Site and Sampling

**Figure 1:**
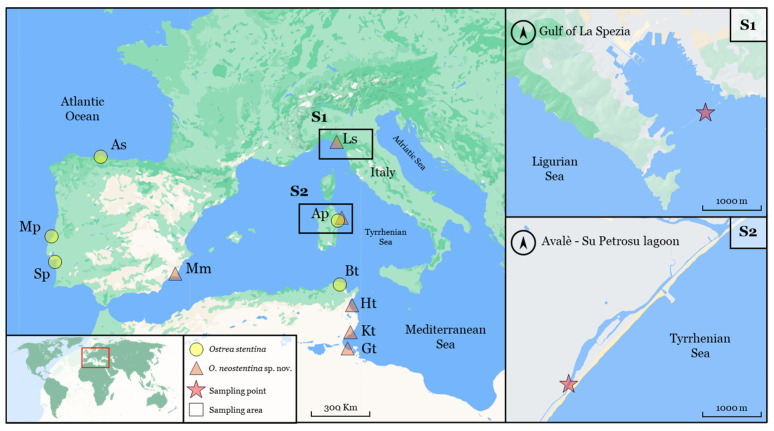
Mediterranean distribution of small flat oysters *Ostrea stentina* and *O. neostentina*. Rectangles denote the study area (Gulf of La Spezia, Liguria (S1), and Sardinia (S2)), while asterisks denote the sampling sites. The data are based on Hu et al. [35], with the addition of Avalè-Su Petrosu and Gulf of La Spezia data from this study. Circles denote Dwarf oyster *O. stentina* (Avilé, Spain (As); Avalè-Su Petrosu Lagoon, Italy (Ap); Bizerte Lagoon, Tunisia (Bt); Mira estuary, Portugal (Mp); Sado estuary, Portugal (Sp)). Triangles denote *O. neostentina* [Avalè-Su Petrosu Lagoon, Italy (Ap); Gannouche, Tunisia (Gt); Gulf of Hammamet, Tunisia (Ht); Kneiss Islands, Tunisia (Kt); Mar Menor lagoon, Spain (Mm); Gulf of La Spezia, Italy (Pv)]. The map from https://mapstyle.withgoogle.com/ (accessed on 3 June 2024) was edited with GIMP 2.10.34 (GNU Image Manipulation Program).

Oysters were collected from natural environments near two shellfish farming areas: the first produces oysters and mussels, while the second focuses solely on the grooved carpet shell, *Ruditapes decussatus*, sourced from natural beds (Figure 1). The first site (a breakwater, (S1); 44°04′27″ N, 09°52′02″ E) is located at the mouth of the Gulf of La Spezia, which cuts it longitudinally between Punta Santa Maria (West) and Punta Santa Teresa (East). The Gulf of La Spezia is a wide and deep inlet along the Ligurian Sea coast, located at the eastern end of the Liguria region. The gulf measures approximately 4.5 km in length and has an average width of 3.5 km.

The other (Avalè-Su Petrosu lagoon (S2); 40°20′39″ N, 09°41′15″ E) is located in the Gulf of Orosei, northeastern Sardinia. Part of the fishery complex called “Cedrino, Avalè-Su Petrosu”, featuring channels and small ponds parallel to the beach, is separated from the Cedrino River by a dune barrier. It also has two sea accesses. The examined lagoon covers an area of approximately 50 hectares, with an average depth of about −2.5 m. Since 2016, burrowing bivalve species, particularly *R. decussatus*, have been harvested in a 9-hectare section of the lagoon.

In early summer 2019 (S1) and 2023 (S2), wild specimens of the genus *Ostrea* spp. were manually sampled from natural beds at a depth of approximately −1 m using a chisel-like tool. A total of 36 specimens were collected, with 12 from S1 and 24 from S2. The specimens were stored dry in a cool box, transported to the laboratory, and kept at −20 °C until molecular analysis.

For each specimen, morphological parameters were measured, including length (mm), width (mm), thickness (mm), weight (g), shell weight (g), and soft tissue weight (g).

A digital caliper with an LCD display (resolution: 0.1 mm, accuracy: ± 0.2 mm) was used for dimensional measurements. The weight measurements were obtained using the VEVOR Digital Analytical Laboratory Balance (precision: 0.01 g).

### 2.2. Molecular Analysis

Genomic DNA was extracted from 25 mg of tissue using the ReliaPrep™ gDNA Tissue Miniprep System (Promega), following the manufacturer’s protocol for animal tissue. The extracted DNA was then quantified with a Qubit™ spectrophotometer using a Qubit™ dsDNA Quantification Broad Range Assay Kit (Thermo Fisher Scientific). A portion of the mitochondrial 16S rRNA gene (16S rRNA) was amplified using the primer pair 16Sar (5′-CGCCTGTTTACCAAAAACAT-3′) and 16Sbr (5′-CC GGTCTGAACTCAGATCACGT-3′) [36]. All reactions were carried out in a volume of 25 µL using 1X FastStart™ PCR Master (Roche), 300 nM of each primer, 250 ng of template, and ultrapure water to the final volume. The thermal cycling protocol was carried out on a Bio-Rad C1000 Touch thermal cycler as follows: 95 °C for 10 min; two cycles at 94 °C for 15 s, at 52 °C for 15 s, at 70 °C for 30 s; two cycles at 94 °C for 15 s, at 48 °C for 15 s, at 70 °C for 30 s; two cycles at 94 °C for 15 s, at 46 °C for 15 s, at 70 °C for 30 s; thirty cycles at 94 °C for 15 s, at 50 °C for 15 s, at 70 °C for 30 s; a final extension at 70 °C for 5 min.

The PCR products were revealed on a 2% agarose gel in TAE buffer. The amplicons were purified using the Extractme DNA clean-up and gel-out kit (Blirt), and 10 ng of template was used as input for cycle sequencing of both strands with the BrilliantDye™ Terminator (v1.1) Cycle Sequencing Kit (Nimagen), following the manufacturer’s instructions. Sequencing was conducted on a SeqStudio Genetic Analyzer capillary sequencer (Thermo Fisher Scientific). The electropherograms were analyzed using SeqMan software (DNASTAR), and the consensus sequence was compared with those present in the GenBank database using the Nucleotide Basic Local Alignment Search Tool (BLASTn) algorithm.

### 2.3. Sequence Data and Phylogenetic Analysis

A 430 bp multiple sequence alignment was built with BioEdit software v. 7.2.5 [37]. The final dataset, comprising n = 35 *Ostrea* spp. sequences and n = 1 common jingle shell *Anomia simplex* sequence from this study, and a single sequence from Bengal whipray *Brevitrygon imbricata* as an outgroup, was utilized to construct a neighbor-joining (NJ) tree based on the 16S rRNA gene. Reference sequences from Hu et al. [35] were used to identify distinct groups in the phylogenetic analysis. The most appropriate nucleotide substitution model (Kimura 2-parameter) was selected by means of jModelTest2 [38] and used as the model input for phylogenetic inference in MEGA v.11 [39]. 

The statistical robustness and reliability of the branching order were confirmed with bootstrap analysis using 1000 reiterations.

All newly generated sequences were deposited in the NCBI GenBank under accession numbers PP178300-PP178334.

### 2.4. Statistical Analysis

All statistical procedures and analyses were performed using R software (v. 4.3.1; R Core Team, 2023). The analyses were applied to the morphometric data of the oyster species. The variables considered included total length (mm), width (mm), thickness (mm), total weight (g), shell weight (g), and soft tissue (g). A level of *p* < 0.05 was set for statistical significance.

Data normality and homoscedasticity were evaluated using the Shapiro–Wilk and the Levene test, respectively (*shapiro.test* and *leveneTest* R-function, respectively). 

The Kruskal–Wallis test by rank (*kruskal.test* R-function) was performed to assess significant differences among the three oyster species (*Ostrea stentina*, *O. neostentina*, and *O. edulis*) for each considered variable. The Dunn test (*dunn.test* R-function) was applied for stochastic dominance and provided results for nonparametric multiple pairwise comparisons following a Kruskal–Wallis test among the three oyster species studied (*k* = 3) [40]. The resulting *p*-values were further adjusted for multiple comparisons using the Bonferroni correction method.

A raincloud plot, generated using the ggplot2 and ggrain R-packages, was employed to illustrate a half-density distribution plot that would improve representation over the conventional boxplot by emphasizing multiple modalities.

Principal Component Analysis (PCA) was also performed (*prcomp* R-function; factoextra R-package) on the data matrix, where rows represented individual oysters and columns represented the morphometric variables described earlier. This approach was used to identify patterns, reduce dimensionality, and highlight the most significant features within the data.

## 3. Results and Discussion

The measurements of the oysters showed that specimens from site 1 (S1; Gulf of La Spezia, Liguria, Italy) had a total length of 91.98 ± 14.49 mm and a total weight of 133.11 ± 56.77 g, while specimens from site 2 (S2; Avalè-Su Petrosu, Sardinia, Italy) had a total length of 43.75 ± 8.35 mm and a total weight of 13.34 ± 8.99 g (Table 1). At S1, the recorded surface water temperature was 18.56 ± 1.25 °C, with a pH of 8.22 ± 0.15, salinity of 37.74 ± 0.96, and dissolved oxygen of 10.36 ± 3.42 mg/L. In contrast, S2 had a higher surface water temperature of 20.24 ± 2.39 °C, a pH of 8.13 ± 0.29, lower salinity at 22.26 ± 10.37, and dissolved oxygen of 9.17 ± 0.76 mg/L.

**Figure 2:**
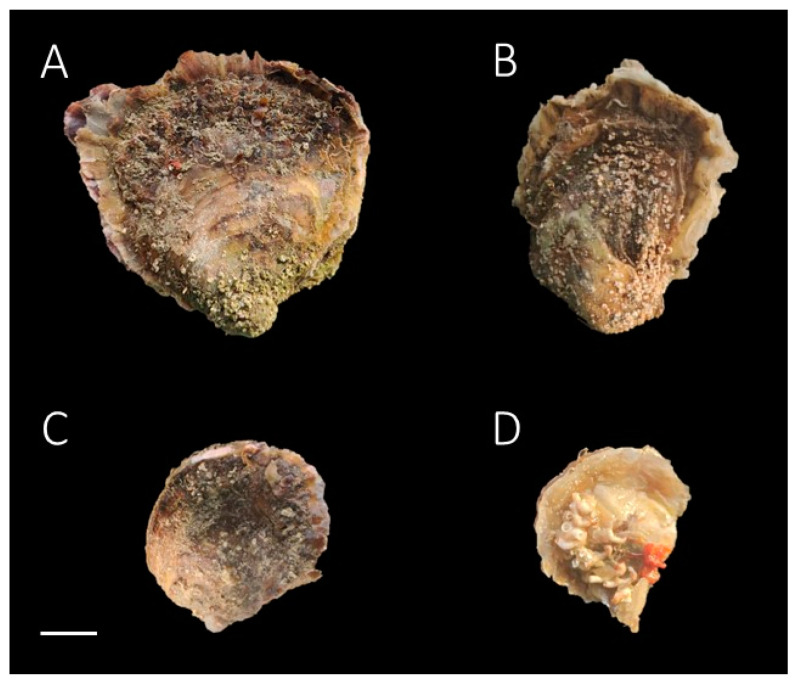
Oysters of the genus *Ostrea*. (**A**,**B**) European flat oyster *Ostrea edulis* from S1 and S2 (Liguria and Sardinia, respectively); (**C**) *O. neostentina* and (**D**) dwarf oyster *O. stentina* from S2. Scale bar: 1 cm. Photography copyright is held exclusively by Giuseppe Esposito and Paolo Pastorino, co-authors of this manuscript.

Several specimens of the oysters are shown in Figure 2A–D. The species affiliation of these specimens, as determined by 16S genotyping and reported in Table 1, indicates that the initial morphological classifications were incorrect in some cases. Figure 2A,B shows a specimen of the European flat oyster *Ostrea edulis* from S1. It has an irregular oval shell with a hooked beak and differently shaped valves. The left valve is deeply concave and fixed, while the right one is flat, serving as a lid. The shell is off-white to cream in colour, with light brown or bluish concentric bands and chalky layers. Figure 2C,D shows *O. neostentina* and the dwarf oyster *O. stentina*, respectively, both sampled from S2. Small, oval oysters with the left valves firmly attached and characterized by raised margins. The right valves are flatter with narrow commissural shelves and finely crenelated edges along the ligament. These observations are consistent with those reported by Hu and co-authors [35]; the morphological data signal the presence of *O. stentina* and *O. neostentina* along the Italian Mediterranean coastline.

Based on shell morphology, 10/12 samples from S1 were attributable to indigenous *O. edulis* (“functionally extinct” in its native range, positively impacting habitat engineering [41]), while the remaining 2/12 were attributable to *O. stentina* (Table 1). At S2, only one specimen of *O. edulis* was found in the natural beds; twenty-one specimens were attributable to *O. stentina* and one specimen to *O. neostentina* [35]. One specimen from S2 was attributed to *Anomia simplex*. To validate species classification based on shell morphology, genetic identification was conducted through phylogenetic analysis of 16S sequencing, which revealed significant differences. Eleven oysters that were classified as *O. stentina* based on shell morphology were actually *O. neostentina* (Table 1). This underscores the crucial role of genetic identification, particularly during the juvenile stages of oysters. Despite morphology’s initial use as a classification method, it may prove insufficient for accurate species discrimination. This becomes especially relevant when considering the notable morphological similarity among species during early developmental stages.

Thus, the results of genetic identification provide crucial insights, highlighting the limitations of relying solely on morphological characteristics for species classification. This underscores the necessity of combining morphological and genetic approaches to achieve accurate species identification, given the observed discrepancies between morphological and genetic classifications.

There was a statistically significant difference (*p* < 0.001) among the average values of the considered variables for the species. Corrections for multiple comparisons highlighted significant differences (adjusted *p* < 0.001) in total length between “*O. edulis*—*O. neostentina*” and “*O. edulis*—*O. stentina*”. For total weight, width, thickness, and soft tissue, no significant differences emerged between “*O. neostentina* and *O. stentina*”. However, the shell weight variable suggested significant differences among the three species.

**Figure 3:**
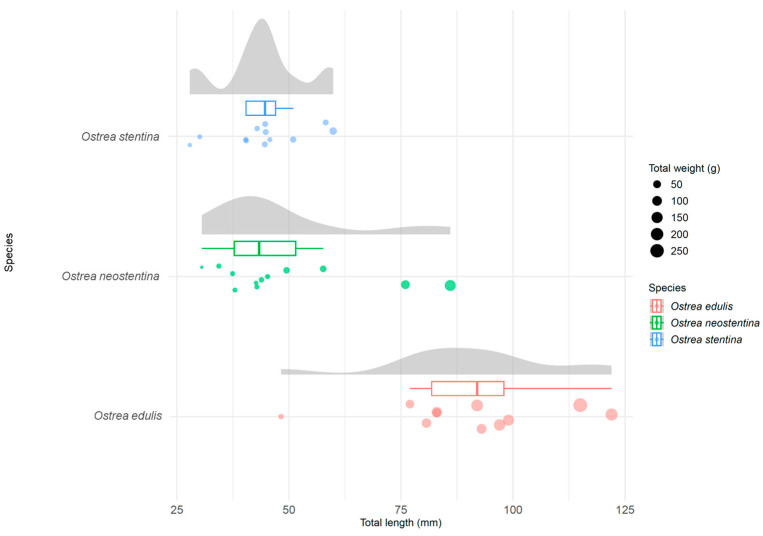
Raincloud plots of the total length of *Ostrea* spp. sampled at the two study sites.

The results, shown through a Raincloud plot, provide a comprehensive representation of morphometric distributions among the species *O. edulis*, *O. neostentina*, and *O. stentina* (Figure 3). The use of box plots, kernel density plots, and swarm plots provides a comprehensive view of central tendencies, dispersion, and distribution shapes, enhancing the understanding of variations in total length among the species.

**Figure 4:**
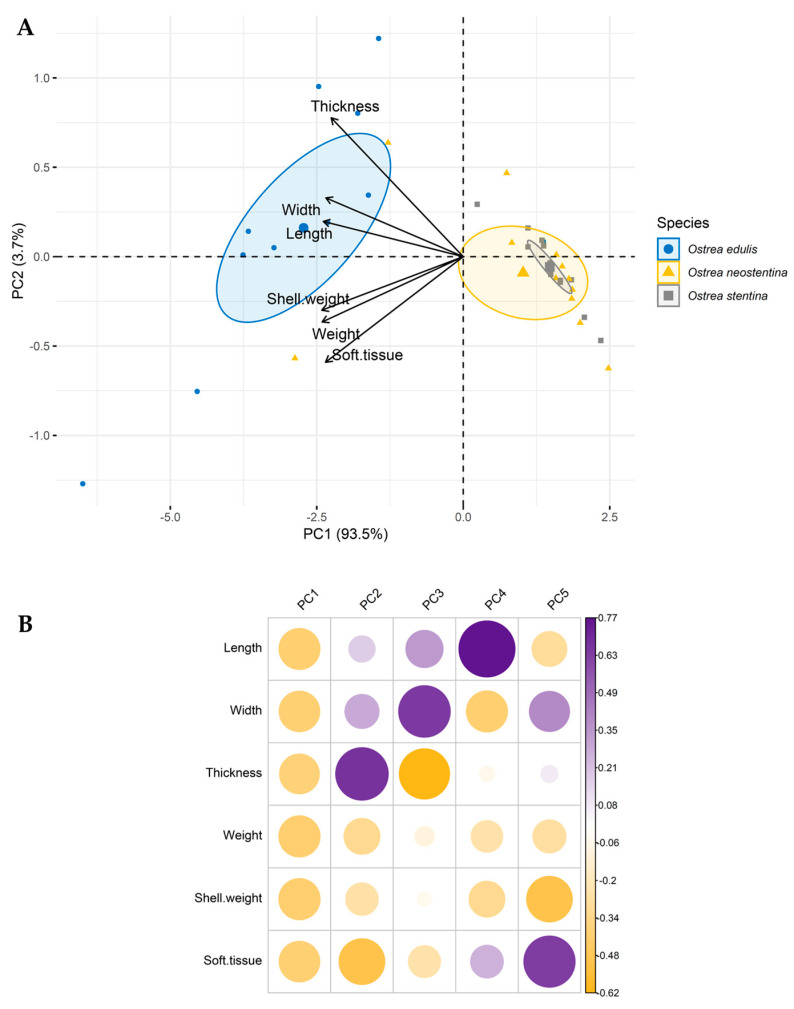
(**A**) Biplot of Principal Component Analysis (PCA) of morphometric data. (**B**) Correlations between the Components and the original variables (the component loadings).

The results of the Principal Component Analysis (PCA) indicate that Component 1 (PC1) significantly explains 93.5% of the total variance, suggesting the presence of crucial information for discriminating among observations (Figure 4). Although Component 2 (PC2) explains a lower percentage (3.7%), it may still contain relevant information. The biplot highlights a tendency for observations of *O. neostentina* and *O. stentina* from S2 to cluster, while two specimens of *O. neostentina* from S1 exhibit proximity to the group of *O. edulis*, also from S1 (Figure 4A). These associations may be linked to size differences and could reflect geographical distinctions. Since our analysis is based on morphometric data, hypotheses regarding the effect of geographic location, environmental variations, or species-specific factors necessitate further investigation or data acquisition for validation. Nevertheless, the limited number of specimens highlighted the impracticality of conducting an in-depth morphological comparison between sites. For example, there were only two specimens of *O. neostentina* in Liguria (S1) and ten in Sardinia (S2). However, in the graphical representation (Figure 1), we focused solely on species-related data, ensuring a detailed and concentrated approach. The component loading plot (Figure 4B) illustrates the correlation coefficients for each variable with respect to each Principal Component, helping to identify which variables have the most significant influence on each component. Overall, PC1 shows relatively similar negative loadings for all variables (approximately −0.4), indicating that this component is uniformly influenced by all variables. PC2 is dominated by thickness, with a high positive loading (0.675), suggesting that this Principal Component is strongly associated with variations in the thickness of the oysters. PC3 is primarily influenced by width (0.652), while PC4 is strongly influenced by length (0.771). The remaining component (PC5) displays mixed contributions, with some variables showing higher correlations than others.

The genus *Ostrea* encompasses both fossil and living species. Harry [27] initially reintroduced the genus *Ostreola* but later reattributed it to *Ostrea* due to the lack of a morphological and genetic basis [27,28,29,31,32].

Several species have been identified as synonyms of *O. stentina* by different researchers, consolidating taxonomic understanding (Hu et al. [35] and references therein). Molecular analysis conducted by Kirkendale and co-authors [29] showed a close phylogenetic relationship between the crested oyster *O. equestris* in the Americas and *O. aupouria* in New Zealand. Subsequent studies suggested that *O. equestris* and *O. aupouria* may be synonyms of *O. stentina*, relying on Mediterranean/eastern Atlantic species [31,42]. 

The taxonomic status of the *O. stentina* species complex, which encompasses various geographies, remains uncertain, necessitating further analysis.

Additionally, the classification of related *Ostrea* species pairs, e.g., *O. edulis* and the Australian mud oyster *O. angasi*, the Olympia oyster *O. conchaphila*, and *O. lurida*, and the sponge oyster *O. permollis* and *O. puelchana* is also debated [26,28,31,32,42,43,44,45,46]. 

In general, small flat oysters have a widespread distribution along the Atlantic Ocean and the Mediterranean Sea. They can also be found along the coasts of North Africa, New Zealand, Japan, China, and South America [30,34,35,47,48].

Recent phylogenetic analysis of the genus *Ostrea* identified four closely related groups within the *O. stentina*/*aupouria*/*equestris* species complex, suggesting they should be recognized as distinct species [35]. The four distinct phylogenetic groups are Group 1 (*O. aupouria*/*stentina* from China, Japan, and New Zealand), which was recommended as *O. equestris* (western Pacific); Group 2 (*O. equestris* from California, Argentina, Florida, and North Carolina), suggested to be classified as *O. equestris* (Americas); Group 3 (*O. stentina* from Mar Menor Lagoon in Spain, eastern Tunisia, Japan, and Hong Kong), described as a new species, *O. neostentina* by Hu et al. [35], and Group 4 (*O. stentina* from Avilés Lagoon in Spain, Portugal, Dakhla Bay in Morocco, and Bizerte Lagoon in northern Tunisia) [35]. Guo and co-authors [26] suggested differentiating the *O. stentina*/*aupouria*/*equestris* complex into three distinct species, including *O. equestris*. 

**Figure 5:**
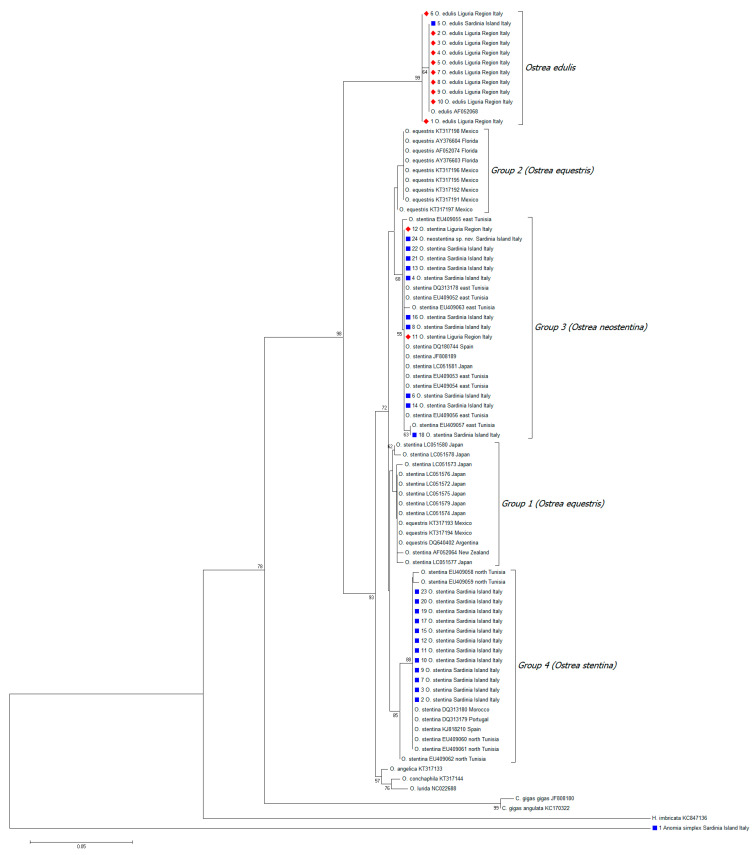
Neighbor-joining (NJ) tree for 35 *Ostrea* spp. sequences and one *Anomia simplex* sequence from this study, along with additional sequences from Hu et al. [35]. Phylogeny was inferred by the alignment of 410 nucleotides. The phylogenetic tree includes sequences from Liguria (S1; red diamond) and Sardinia (S2; blue square). Bootstrap (1000 replicates) values over 50 are shown at the internal nodes. The length of each pair of branches represents the distance between sequence pairs. The scale bar represents the percentage of nucleotide differences.

Phylogenetic analysis of 16S sequence data from this study is consistent with the findings of Hu et al. [35]. Our phylogenetic tree reproduced the four main clusters (Groups 1–4), although none of our sequences clustered in Groups 1 and 2 (both *O. equestris*) (Figure 5). Group 3 (*O. neostentina*) included 10/24 sequences from S2 and 2/12 sequences from S1. Group 4 (*O. stentina*) included 12/24 sequences from S2. The remaining oyster specimens clustered in the *O. edulis* group (n = 10 (S1) and n = 1 (S2)).

Looking at the 16S diversity within species, we observed a unique haplotype for *O. stentina* and two haplotypes for both *O. neostentina* and *O. edulis*; the minor haplotypes differed by only one nucleotide in the analyzed 16S region and occurred in 1/12 (SNP A > G) and 2/11 (SNP C > T) sequences of *O. neostentina* and *O. edulis*, respectively. The 16S genetic distance among the three species ranged from 2.1% between *O. stentina* and *O. neostentina*, up to 7.8% between *O. edulis* and *O. stentina* and 7.9% between *O. edulis* and *O. neostentina*. The genetic distance between *O. stentina* and *O. neostentina* observed in this study is higher than what has been previously reported for this species pair (1.2–1.7% [35]) and is similar to that observed between the Portuguese oyster *Crassostrea angulata* and the Kumamoto oyster *C. sikamea* (2.1%, [35,49]). The observed genetic distance supports the classification of *O. neostentina* as a separate species from *O. stentina*, as the intraspecific distance in 16S is significantly lower than (1% [35,49]). The classification of *O. neostentina* is supported by mitochondrial sequence data, including 16S rRNA, as detailed by Hu et al. [35]. While our study provides a substantial basis for this classification, further research is needed to confirm and better understand this new species.

The distribution and abundance of *O. neostentina* are not well understood. The finding that *O. neostentina* accounts for 50% of the oysters sampled from S2 in this study suggests it might be common in the Gulf of Orosei, northeastern Sardinia, Italy. If so, this region provides a good area for studying the biology of this species that is largely unknown. The finding of *O. neostentina* at S2, where salinity varies greatly (22.26 ± 10.37), suggests that it may tolerate a wide range of salinities. 

The morphological variability in oysters is a source of taxonomic confusion, impacting aquaculture and management; however, molecular analysis can help clarify oyster identities and evolutionary backgrounds, as demonstrated in this and other studies [26,34,46,50,51].

Oyster farming is a significant component of global aquaculture production, contributing to sustainable aquatic food supplies [4].

Bivalves, including small flat oysters, offer excellent nutritional benefits with minimal environmental impact compared to other livestock [52,53]. Identifying undocumented oyster species in the Mediterranean could enhance production and conservation efforts, as demonstrated by recent studies on the feasibility of hatchery and nursery production of *O. stentina* for conservation purposes [54].

## 4. Conclusions

This study adds the current knowledge of the distribution of small flat oysters within the *Ostrea stentina* species complex along the Italian Mediterranean coast. Our findings provide the first documented evidence of *O. neostentina* in Liguria and Sardinia (Italy), expanding the known range of this species. This new data is crucial for assessing the ecological dynamics and conservation status of *O. neostentina*.

Further research is essential to monitor the distribution and population dynamics of these oysters, as well as to develop and implement effective management protocols. Such efforts will be vital for ensuring the sustainability of their populations and addressing potential conservation concerns. Additionally, understanding the ecological roles of these oysters in their habitats could offer insights into the broader marine ecosystem’s health and resilience.

## Figures and Tables

**Table 1:** Species identity and morphometrics of oysters sampled at S1 (Liguria) and S2 (Sardinia).

Site	No.	Species (Morphology)	Species (After Phylogeny)	Total Length (mm)	Width (mm)	Thickness (mm)	Total Weight (g)	Shell Weight (g)	Soft Tissue (g)
Liguria S1	1	*Ostrea edulis*	*O. edulis*	80.7	81	25	90.8	76.04	6.39
	2	*O. edulis*	*O. edulis*	99	77	28	140.51	109.73	16.57
	3	*O. edulis*	*O. edulis*	122	91	23	184.69	147.27	22.65
	4	*O. edulis*	*O. edulis*	115	86	37	263.69	199.85	35.62
	5	*O. edulis*	*O. edulis*	97	79	32	157.89	118.59	18.12
	6	*O. edulis*	*O. edulis*	92	71	34	171.92	143.84	17.22
	7	*O. edulis*	*O. edulis*	77	70	30	69.23	60.09	5.76
	8	*O. edulis*	*O. edulis*	83	76	25	115.91	94.45	11.88
	9	*O. edulis*	*O. edulis*	83	61	26	79.54	65.02	12.37
	10	*O. edulis*	*O. edulis*	93	66	34	102.24	84.93	11.16
	11	*O. stentina*	*O. neostentina*	76	57	28	74.72	59.39	9.26
	12	*O. stentina*	*O. neostentina*	86	80	21	146.19	120.56	16.03
Sardinia S2	1	*Anomia simplex*	*A. simplex*	49.17	45.82	7.02	8.38	6.75	1.28
	2	*O. stentina*	*O. stentina*	59.87	46.22	19.04	42.68	31.95	4.47
	3	*O. stentina*	*O. stentina*	50.96	42.86	12.81	20.80	16.04	2.02
	4	*O. stentina*	*O. neostentina*	57.65	45.66	13.05	27.95	21.26	2.32
	5	*O. edulis*	*O. edulis*	48.24	39.47	12.52	12.04	10.26	1.26
	6	*O. stentina*	*O. neostentina*	49.47	44.63	18.91	26.45	22.31	2.07
	7	*O. stentina*	*O. stentina*	58.20	41.91	12.55	16.64	15.26	0.96
	8	*O. stentina*	*O. neostentina*	43.87	34.52	11.23	12.85	11.37	1.26
	9	*O. stentina*	*O. stentina*	42.91	32.59	11.17	11.78	10.39	1.18
	10	*O. stentina*	*O. stentina*	44.70	37.14	14.07	15.88	12.65	1.63
	11	*O. stentina*	*O. stentina*	44.61	37.80	11.58	16.22	12.22	0.81
	12	*O. stentina*	*O. stentina*	44.83	38.09	13.13	17.64	13.71	1.01
	13	*O. stentina*	*O. neostentina*	42.85	32.30	11.79	8.99	7.90	0.84
	14	*O. stentina*	*O. neostentina*	42.66	32.01	6.79	4.73	3.23	0.48
	15	*O. stentina*	*O. stentina*	40.36	30.90	10.82	9.76	2.17	0.69
	16	*O. stentina*	*O. neostentina*	37.95	35.26	9.02	7.60	6.68	0.74
	17	*O. stentina*	*O. stentina*	45.74	41.07	10.09	7.80	8.81	1.81
	18	*O. stentina*	*O. neostentina*	34.36	33.06	10.67	9.82	6.26	0.90
	19	*O. stentina*	*O. stentina*	40.47	35.54	10.94	10.67	8.48	1.37
	20	*O. stentina*	*O. stentina*	30.10	32.15	8.80	6.90	4.77	0.81
	21	*O. stentina*	*O. neostentina*	37.42	30.72	11.78	9.19	7.02	1.05
	22	*O. stentina*	*O. neostentina*	45.24	31.13	13.07	9.90	8.51	1.21
	23	*O. stentina*	*O. stentina*	27.88	23.26	8.37	3.72	3.07	0.50
	24	*O. neostentina*	*O. neostentina*	30.55	22.33	5.75	1.83	1.29	0.20

## Data Availability

Data are available on request.
